# First Month of COVID-19 Vaccine Safety Monitoring — United States, December 14, 2020–January 13, 2021

**DOI:** 10.15585/mmwr.mm7008e3

**Published:** 2021-02-26

**Authors:** Julianne Gee, Paige Marquez, John Su, Geoffrey M. Calvert, Ruiling Liu, Tanya Myers, Narayan Nair, Stacey Martin, Thomas Clark, Lauri Markowitz, Nicole Lindsey, Bicheng Zhang, Charles Licata, Amelia Jazwa, Mark Sotir, Tom Shimabukuro

**Affiliations:** ^1^CDC COVID-19 Response Team; ^2^Food and Drug Administration, Silver Spring, Maryland.

Two coronavirus disease 2019 (COVID-19) vaccines are currently authorized for use in the United States. The Food and Drug Administration (FDA) issued Emergency Use Authorization (EUA) for the Pfizer-BioNTech COVID-19 vaccine on December 11, 2020, and for the Moderna COVID-19 vaccine on December 18, 2020; each is administered as a 2-dose series. The Advisory Committee on Immunization Practices issued interim recommendations for Pfizer-BioNTech and Moderna COVID-19 vaccines on December 12, 2020 (*1*), and December 19, 2020 (*2*), respectively; initial doses were recommended for health care personnel and long-term care facility (LTCF) residents (*3*). Safety monitoring for these vaccines has been the most intense and comprehensive in U.S. history, using the Vaccine Adverse Event Reporting System (VAERS), a spontaneous reporting system, and v-safe,* an active surveillance system, during the initial implementation phases of the COVID-19 national vaccination program (*4*). CDC conducted descriptive analyses of safety data from the first month of vaccination (December 14, 2020–January 13, 2021). During this period, 13,794,904 vaccine doses were administered, and VAERS received and processed^†^ 6,994 reports of adverse events after vaccination, including 6,354 (90.8%) that were classified as nonserious and 640 (9.2%) as serious.^§^ The symptoms most frequently reported to VAERS were headache (22.4%), fatigue (16.5%), and dizziness (16.5%). A total of 113 deaths were reported to VAERS, including 78 (65%) among LTCF residents; available information from death certificates, autopsy reports, medical records, and clinical descriptions from VAERS reports and health care providers did not suggest any causal relationship between COVID-19 vaccination and death. Rare cases of anaphylaxis after receipt of both vaccines were reported (4.5 reported cases per million doses administered). Among persons who received Pfizer-BioNTech vaccine, reactions reported to the v-safe system were more frequent after receipt of the second dose than after the first. The initial postauthorization safety profiles of the two COVID-19 vaccines in current use did not indicate evidence of unexpected serious adverse events. These data provide reassurance and helpful information regarding what health care providers and vaccine recipients might expect after vaccination.

VAERS is an existing national passive surveillance system for adverse events after vaccination that accepts reports from health care providers, vaccine manufacturers, and the public. Reported signs and symptoms are coded using Medical Dictionary for Regulatory Activities (MedDRA) terminology.^¶^ Serious adverse events are followed up by the VAERS program to obtain additional information, including medical records, information from health care providers, and, in the case of death, death certificates and autopsy reports (*4*).

V-safe is a safety monitoring system established by CDC specifically for the COVID-19 vaccination program. V-safe participants voluntarily self-enroll and receive smartphone text messages providing hyperlinks to web surveys.** During the first week after vaccination, enrollees complete daily surveys asking about local injection site and systemic reactions. Enrollees are asked if they missed work, were unable to perform normal daily activities, or received care from a medical professional because of reported symptoms or heath conditions. Enrollees who report seeking medical care are contacted, and a VAERS report is completed if clinically indicated. Persons who do not report their sex as male are asked about pregnancy status at time of vaccination (initial survey) and about a positive pregnancy test result (3- and 6-week surveys); reported pregnancies are followed up through the v-safe pregnancy registry.^††^

CDC conducted descriptive analyses of data from VAERS and v-safe during December 14, 2020–January 13, 2021, a period when the first and second doses of Pfizer-BioNTech vaccine and the first dose of Moderna vaccine were administered. Because LTCF staff members were vaccinated at LTCF facilities, residents of LTCFs were presumptively identified by restricting examination of VAERS reports to adults aged ≥65 years with a documented vaccination at an LTCF. To ensure that LTCF residents with serious adverse events were identified, manual review was conducted of all reports of serious adverse events among those vaccinated in LTCFs, regardless of vaccine recipient’s age. Administered vaccine doses were reported to CDC.^§§^ These activities were reviewed by CDC and are consistent with applicable federal law and CDC policy.^¶¶^All analyses were conducted using SAS software (version 9.4; SAS Institute).

During December 14, 2020–January 13, 2021, a total of 13,794,904 COVID-19 vaccine doses were administered in the United States; 8,436,863 (61.2%) doses were administered to women. VAERS received 6,994 reports of COVID-19–associated adverse events during this period. Among all reports, 6,354 (90.8%) were classified as nonserious and 640 (9.2%) as serious, including 113 (1.6%) deaths. The median age of vaccine recipients in VAERS reports was 42 years (range = 15–104 years); 5,505 (78.7%) reports were submitted for adverse events in women. Headache (22.4%), fatigue (16.5%), and dizziness (16.5%) were the most frequently reported symptoms after vaccination with either vaccine (Table 1). Sixty-two reports of anaphylaxis have been confirmed, 46 (74.2%) after receipt of the Pfizer-BioNTech vaccine and 16 (25.8%) after receipt of the Moderna vaccine.

**TABLE 1:** Reports of adverse events after receipt of Pfizer-BioNTech and Moderna COVID-19 vaccines, by recipients’ demographic characteristics and reported symptoms — Vaccine Adverse Event Reporting System, United States, December 14, 2020–January 13, 2021

Characteristic	No. (%) reporting adverse events			
	All COVID-19 vaccine doses (N = 6,994)	Pfizer-BioNTech vaccine		Moderna vaccine Dose 1 (N = 1,373)
		Dose 1 (N = 5,428)	Dose 2 (N = 193)	
**Nonserious adverse event reports**	6,354 (90.9)	5,087 (93.7)	152 (78.6)	1,115 (81.2)
Serious adverse event reports*^†^	640 (9.2)	341 (6.3)	41 (21.2)	258 (18.8)
**Sex**				
Female	5,505 (78.7)	4,296 (79.2)	142 (73.6)	1,067 (77.7)
Male	1,408 (20.1)	1,056 (19.5)	51 (26.4)	301 (21.9)
Unknown	81 (1.2)	76 (1.4)	0 (—)	5 (0.4)
**Age group (yrs)**				
0–17	12 (0.2)	4 (0.1)	0 (—)	8 (0.6)
18–49	4,539 (64.9)	3,568 (65.7)	119 (61.7)	852 (62.1)
50–64	1,772 (25.3)	1,351 (24.9)	51 (26.4)	370 (27.0)
65–74	255 (3.7)	184 (3.4)	11 (5.7)	60 (4.4)
75–84	85 (1.2)	48 (0.9)	5 (2.6)	32 (2.3)
≥85	93 (1.3)	46 (0.9)	4 (2.1)	43 (3.1)
Unknown	238 (3.4)	227 (4.2)	3 (1.6)	8 (0.1)
**Most frequently reported symptoms**				
Headache	1,566 (22.4)	1,184 (21.8)	35 (18.1)	347 (25.3)
Fatigue	1,154 (16.5)	912 (16.8)	14 (7.3)	228 (16.6)
Dizziness	1,151 (16.5)	907 (16.7)	16 (8.3)	228 (16.6)
Chills	1,040 (14.9)	760 (14.0)	19 (9.8)	261 (19.0)
Nausea	1,037 (14,8)	790 (14.6)	18 (9.3)	229 (16.7)

**Abbreviation:** COVID-19 = coronavirus disease 2019.

* Based on the Code of Federal Regulations, classification of a serious adverse event includes a report of one of the following: death, life-threatening illness, hospitalization or prolongation of hospitalization, permanent disability, congenital anomaly, or birth defect. https://www.accessdata.fda.gov/scripts/cdrh/cfdocs/cfcfr/cfrsearch.cfm?fr

^†^ Includes 113 deaths.

## VAERS Reports Involving Non-LTCF Residents

Among the 6,994 VAERS reports received and processed, 6,844 (97.9%) involved persons not residing in LTCFs; among these, 5,533 (80.8%) received the Pfizer-BioNTech vaccine and 1,311 (19.2%) received the Moderna vaccine. Most reports concerned women (5,413; 79.1%), and the median age of persons reporting adverse events was 42 years (range = 15–96 years). The most frequently reported symptoms were headache (1,564; 22.9%), dizziness (1,149; 16.8%), and fatigue (1,147; 16.8%). Among these reports, 6,326 (92.4%) were classified as nonserious. Included among the 518 (7.6%) serious reports were 35 reports of death: 16 (45.7%) after the Pfizer-BioNTech vaccine and 19 (54.3%) after the Moderna vaccine. Decedents ranged in age from 25 to 91 years (median = 62 years); 15 (42.9%) were women. The median interval from vaccination to death was 3 days (range = 0–20 days). Among 19 persons whose deaths were reported to VAERS after receiving COVID-19 vaccine, record collection and evaluation are ongoing; for the remaining 16 reported deaths, review of death certificates or other data indicated underlying heart disease, cancer, stroke, probable pulmonary embolism, and otherwise frail health as the cause of death.

## VAERS Reports Involving LTCF Residents

Among residents of LTCFs who received COVID-19 vaccine, 150 (2.1%) reports of adverse events were submitted to VAERS, including 88 (58.7%) after receipt of the Pfizer-BioNTech vaccine and 62 (41.3%) after receipt of the Moderna vaccine. The median vaccine recipient age was 83 years (range = 17–104 years), and 92 (61%) reports concerned women. Among 122 (81.3%) reports of serious adverse events in LTCF residents, 78 (52.0%) deaths have been reported and investigated; 42 (53.8%) occurred in residents in hospice care or with a do-not-resuscitate status. Death certificate data were available for 17 (22.0%) deaths; causes of death included cardiac disease, dementia, pneumonia, and failure to thrive. Nineteen (24.3%) reported deaths are currently awaiting additional records to establish cause of death. Reported deaths occurred 0–20 days after vaccination (median = 2 days).

## v-safe Reports

During December 14, 2020–January 13, 2021, v-safe enrolled 1,602,065 vaccine recipients who completed at least one survey; 814,648 (50.8%) received Pfizer-BioNTech, and 787,417 (49.2%) received Moderna vaccines. The median recipient age was 46 years (range = 16–110 years); 1,106,656 (69.1%) were women. There were 10,825 (0.68%) enrollees who reported that they were pregnant at the time of vaccination, and 262 (0.02%) reported a positive pregnancy test result after vaccination. Solicited local and systemic reactions were similar between persons receiving first doses of Pfizer-BioNTech and Moderna vaccines. Injection site pain, fatigue, headache, myalgia, and chills were most frequently reported (Figure). Enrollees reported more reactions on the day after vaccination than on any other day. For the Pfizer-BioNTech vaccine, reactions were more frequent after the second dose than the first; the reported rate of fever and chills was more than four times higher after the second dose than after the first (Table 2).

**FIGURE:**
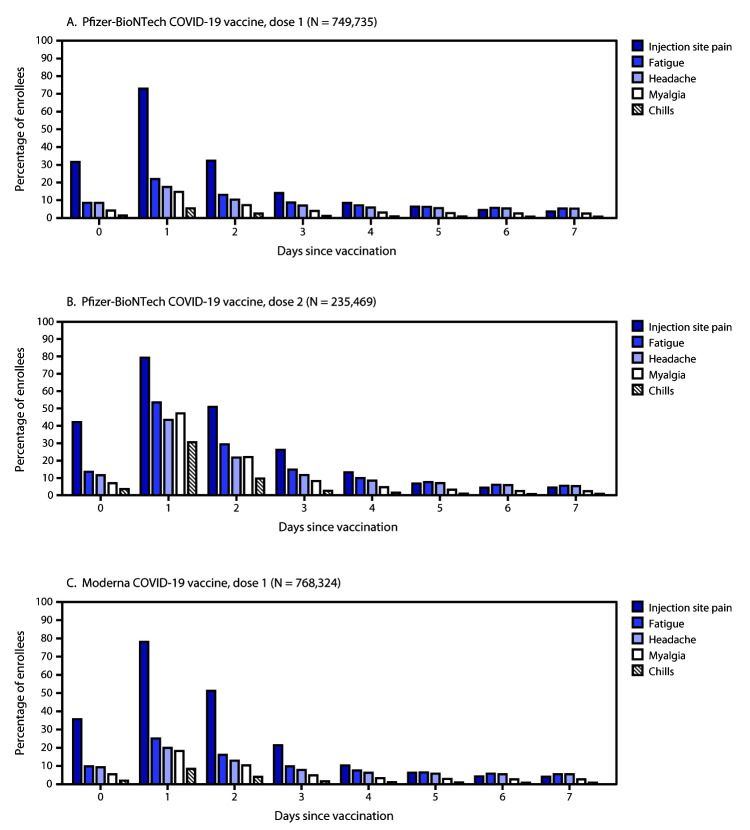
Percentage of enrollees who reported common local and systemic reactions by day after receipt of the first dose of Pfizer BioNTech COVID-19 vaccine (A), second dose of Pfizer BioNTech COVID-19 vaccine (B), and first dose of Moderna COVID-19 vaccine (C) — v-safe, United States, December 14, 2020–January 13, 2021 **Abbreviation:** COVID-19 = coronavirus disease 2019.

**TABLE 2:** Percentage of v-safe enrollees who completed at least one survey (N = 1,602,065) with local and systemic reactions reported for day 0–7 and for day 1 after receiving Pfizer-BioNTech and Moderna COVID-19 vaccines — v-safe,* United States, December 14, 2020–January 13, 2021

Local and systemic reaction	Percentage of v-safe enrollees reporting reactions			
	Both vaccines	Pfizer-BioNTech vaccine		Moderna vaccine
	Day 0–7	Dose 1, day 1	Dose 2, day 1	Dose 1, day 1
Injection site pain	70.9	72.9	79.3	78.1
Fatigue	33.5	21.9	53.5	25.1
Headache	29.5	17.5	43.4	19.9
Myalgia	22.9	14.7	47.2	18.3
Chills	11.6	5.5	30.6	8.4
Fever	11.4	5.8	29.2	8.2
Injection site swelling	10.8	6.2	8.6	12.6
Joint pain	10.4	5.3	23.5	7.3
Nausea	8.9	4.2	14.0	5.5

**Abbreviation:** COVID-19 = coronavirus disease 2019.

* https://www.cdc.gov/coronavirus/2019-ncov/vaccines/safety/vsafe.html

## Discussion

After administration of 13.8 million doses of Pfizer-BioNTech and Moderna COVID-19 vaccines to the U.S. population during the first month of the vaccination program, the postauthorization safety profiles for both vaccines are reassuring. Most (90.9%) VAERS reports were for nonserious events and involved local and systemic symptoms; transient local and systemic reactions were also frequently reported in v-safe. Reports of anaphylaxis have been observed after administration of both vaccines (*5*). The occurrence of anaphylaxis after receipt of COVID-19 vaccines during the analytic period, 4.5 cases per million doses administered, is within the range reported after receipt of inactivated influenza vaccine (1.4 per million), pneumococcal polysaccharide vaccine (2.5 per million), and live attenuated herpes zoster vaccine (9.6 per million); effective treatments for anaphylaxis exist (*6*).

VAERS received 113 reports of death after COVID-19 vaccinations; two thirds of these deaths occurred among LTCF residents. All-cause mortality is high in LTCF populations because underlying medical conditions are common. Based on expected rates of background mortality, among the approximately 1 million LTCF residents vaccinated in the first month of the U.S. COVID-19 vaccination program, approximately 7,000 coincidental, temporally associated deaths from all causes would be expected during the analytic period (*7*). In contrast, VAERS received 78 reports of death after COVID-19 vaccination in LTCF residents, and approximately one half were in residents who were in hospice or who had a do-not-resuscitate status. Reported causes of death in LTCF residents after COVID-19 vaccination are consistent with expected all-cause mortality in this population. Among deaths in persons with available death certificate and autopsy information who were not LTCF residents, causes of death were consistent with background all-cause mortality and did not indicate any unexpected pattern that might suggest a causal relationship with vaccination (*8*).

Findings from v-safe monitoring for both vaccines indicate substantial reactogenicity. More reactogenicity was reported after the second dose of Pfizer-BioNTech than the first, particularly on the day after vaccination (data on second dose of Moderna vaccine were not available because of later availability and the dosing interval). These findings are similar to those from clinical trials from both manufacturers, in which injection site pain, fatigue, headache, and myalgia were most frequently reported, with a higher frequency after the second dose in comparable age groups (*9*,*10*). V-safe’s rapid collection of experiences from vaccinated persons provides valuable information that health care providers can use to counsel vaccine recipients about common reactions and what to expect after vaccination.*** V-safe will be able to provide information on vaccination during pregnancy through follow-up in the v-safe pregnancy registry.

The findings in this report are subject to at least three limitations. First, VAERS analyses are based on passive surveillance, and reporting biases are possible, both from underreporting because of lack of awareness or compliance with reporting requirements as well as from stimulated reporting related to increased awareness. Second, LTCF residents might have been undercounted because the search strategy for identifying LTCF residents relied primarily on vaccination facility documentation. Because of challenges in distinguishing LTCF staff members from LTCF residents aged ≤65 years, only serious VAERS reports were reviewed among those aged ≤65 years who were vaccinated in LTCFs. Finally, v-safe is a voluntary self-enrollment program requiring smartphone access, and all vaccination locations might not have offered equal access to v-safe enrollment materials to vaccine recipients; therefore, information from v-safe might not be representative or generalizable.

Mass vaccination with highly effective vaccines is critical to controlling the COVID-19 pandemic. Because of the speed of COVID-19 vaccine development and deployment, there have been concerns among the public about the safety of these new vaccines. In response to these concerns, the U.S. government has implemented the most comprehensive vaccine safety monitoring program in its history. Cases of anaphylaxis after receipt of both authorized vaccines have been observed, though rarely; anaphylaxis rates are comparable with those reported after receipt of other vaccines. No unexpected patterns of reactions or other safety concerns have been identified during early monitoring. CDC and FDA will continue to monitor the safety of COVID-19 vaccines to inform vaccination policy and to maintain public confidence.

Adverse events that occur after COVID-19 vaccination should be reported to VAERS. Providers are encouraged to promote v-safe enrollment and are required under EUA to report to VAERS vaccination administration errors, serious adverse events, cases of multisystem inflammatory syndrome, and cases of COVID-19 that result in hospitalization or death after COVID-19 vaccination.^†††^ These initial findings should provide reassurance to health care providers and to vaccine recipients and promote confidence in the safety of COVID-19 vaccines.

**Summary**

**What is already known about this topic?**

Two COVID-19 vaccines have received Emergency Use Authorization for administration in the United States. In preauthorization clinical trials, local and systemic reactions were reported; no serious safety problems were detected.

**What is added by this report?**

Monitoring, conducted as part of the U.S. vaccination program, indicates reassuring safety profiles for COVID-19 vaccines. Local and systemic reactions were common; rare reports of anaphylaxis were received. No unusual or unexpected reporting patterns were detected.

**What are the implications for public health practice?**

Health care providers and vaccine recipients can be reassured about the safety of Pfizer BioNTech and Moderna COVID-19 vaccines. Counseling vaccine recipients to expect transient local and systemic reactions might ease concerns and encourage completion of the 2-dose vaccination series.

## Footnotes

*
https://www.cdc.gov/coronavirus/2019-ncov/vaccines/safety/vsafe.html

† Processed VAERS reports are those that have been MedDRA-coded, been deduplicated, and undergone quality assurance and quality control.

§ Based on the Code of Federal Regulations, a serious adverse event is defined as occurring if one of the following is reported: death, life-threatening illness, hospitalization or prolongation of hospitalization, permanent disability, congenital anomaly, or birth defect. https://www.accessdata.fda.gov/scripts/cdrh/cfdocs/cfcfr/cfrsearch.cfm?fr

¶ A single VAERS report might be assigned more than one MedDRA Preferred Term; not all terms are medically confirmed diagnoses. https://www.meddra.org/how-to-use/basics/hierarchy

** V-safe enrollees receive daily health check-ins via smartphone text messages that link to web-based surveys for the first 7 days after vaccination, then weekly through 6 weeks postvaccination and then at 3, 6, and 12 months. The health check-in process resets when a person receives a second dose of vaccine.

††
https://www.cdc.gov/vaccinesafety/pdf/vsafe-pregnancy-surveillance-protocol-508.pdf

§§
https://covid.cdc.gov/covid-data-tracker/#datatracker-home

¶¶ 45 C.F.R. part 46, 21 C.F.R. part 56; 42 U.S.C. Sect. 241(d); 5 U.S.C. Sect. 552a; 44 U.S.C. Sect. 3501 et seq.

***
https://www.cdc.gov/vaccines/covid-19/info-by-product/clinical-considerations.html

†††
https://vaers.hhs.gov/faq.html
